# Proteomic Response of *Pseudomonas aeruginosa* PAO1 Adhering to Solid Surfaces

**DOI:** 10.3389/fmicb.2017.01465

**Published:** 2017-08-03

**Authors:** Morgan Guilbaud, Jérôme Bruzaud, Emeline Bouffartigues, Nicole Orange, Alain Guillot, Anne Aubert-Frambourg, Véronique Monnet, Jean-Marie Herry, Sylvie Chevalier, Marie-Noëlle Bellon-Fontaine

**Affiliations:** ^1^Micalis Institute, INRA, AgroParisTech, Université Paris-Saclay Jouy-en-Josas, France; ^2^Laboratoire de Microbiologie, Signaux et Microenvironnement, Normandie Université, Université de Rouen-Normandie Rouen, France

**Keywords:** *Pseudomonas aeruginosa*, adhesion, abiotic material, physicochemical properties, hydrophobicity, roughness, proteome modification, porins

## Abstract

*Pseudomonas aeruginosa* is a pathogenic micro-organism responsible for many hospital-acquired infections. It is able to adhere to solid surfaces and develop an immobilized community or so-called biofilm. Many studies have been focusing on the use of specific materials to prevent the formation of these biofilms, but the reactivity of the bacteria in contact to surfaces remains unknown. The aim of this study was to evaluate the impact of the abiotic surface on the physiology of adherent bacteria. Three different materials, stainless steel (SS), glass (G), and polystyrene (PS) that were relevant to industrial or medical environments were characterized at the physicochemical level in terms of their hydrophobicity and roughness. We showed that SS was moderately hydrophilic and rough, potentially containing crevices, G was hydrophilic and smooth while PS was hydrophobic and smooth. We further showed that *P. aeruginosa* cells were more likely able to adhere to SS and G rather than PS surfaces under our experimental conditions. The physiological response of *P. aeruginosa* when adhering to each of these materials was then evaluated by global proteomic analysis. The abundance of 70 proteins was shown to differ between the materials suggesting that their abundance was modified as a function of the material to which bacteria adhered. Our data lead to enabling the identification of abundance patterns that appeared to be specific to a given surface. Taken together, our data showed that *P. aeruginosa* is capable of sensing and responding to a surface probably *via* specific programmes to adapt its physiological response accordingly.

## Introduction

*Pseudomonas aeruginosa* is an opportunistic pathogen responsible for a variety of human infections such as pneumonia, aggravated cystic fibrosis, septic shock, urinary tract infection, skin, and soft tissue infections. It is also involved in nosocomial infections due to the development of resistance mechanisms to disinfecting procedures, notably in healthcare environments. Various surfaces can be contaminated by this pathogen and may become new starting points for its dissemination (Dunne, 2002; Leibovitz et al., 2003; Zegans et al., 2005). The exceptional survivability properties of *P. aeruginosa* have been related to this strain's ability to colonize solid substrates and form three-dimensional (3D) structured aggregates of bacterial cells called biofilms (Costerton et al., 1995). In this state, the bacteria become immobilized and embedded into an exopolymer-containing matrix, leading to their increased resistance to antimicrobials, either *via* the chemical and mechanical protections provided by the matrix itself, or by the considerable physiological modifications that are encountered by the bacteria in these conditions compared to motile planktonic cells (Stewart and Costerton, 2001; Whiteley et al., 2001; Drenkard and Ausubel, 2002). Many studies have been performed on sessile cells at the proteomic level (Sauer et al., 2002) and showed that the bacterial protein content was markedly modified, depending on various parameters such as nutritional cues (carbon and nitrogen sources, calcium, or ferrous ion concentrations), temperature, oxygen availability, or even biofilm maturity (Whiteley et al., 2001; Sauer et al., 2002; Yoon et al., 2002; Arevalo-Ferro et al., 2003; Vilain et al., 2004; Southey-Pillig et al., 2005; Waite et al., 2005; Patrauchan et al., 2007). While many studies have focused on the physiology of the cells within biofilms, few have examined their behavior during the first stage of biofilm formation, i.e., adhesion. To date, the impact of the material on bacterial physiology during the adhesion stage is far from being known. Bacterial adhesion to inert surfaces has been shown to impact the viability of cells (Terada et al., 2006; Nejadnik et al., 2008), their resistance to antibiotics (Aaron et al., 2002) and their virulence (Siryaporn et al., 2014). Most studies of bacterial adhesion have hitherto only focused on the amount of adherent cells found on various abiotic surfaces (Yuan et al., 2011; Gadenne et al., 2013; Bedel et al., 2015) in relation to the surface energy, charge, topography, and stiffness of the materials employed (Song et al., 2015). Surface stiffness was recently shown to impact the proteome of a non-pathogenic strain of *Pseudoalteromonas* sp. when adhering to agar (Guégan et al., 2013), suggesting that bacteria are able to sense different surfaces (Busscher and van der Mei, 2012; Song et al., 2015). Remarkably, in *P. aeruginosa* the two SadC (surface attachment defective) and Wsp (wrinkly spreader phenotype) regulated circuits have been shown to be involved in surface sensing, leading to higher production of the second messenger bis-(3′–5′)-cyclic guanosine monophosphate (c-di-GMP) resulting in biofilm formation (Belas, 2014). However, the degree to which the physiology of adhering bacteria is affected by the physicochemical properties of the material involved still needs to be elucidated. This is a key question, particularly with respect to safety issues, because the answers would enable strategies to optimize the control of surface bio-contamination (choice of material, modification of the solid surface, etc.) and consequently to reduce associated risks. The aim of this study was therefore to evaluate the impact of the abiotic surface on the physiology of adherent bacteria. Three different materials (stainless steel, glass, and polystyrene) that were relevant to industrial or medical environments were first of all characterized at the physicochemical level in terms of their hydrophobicity and roughness. The physiological response of *P. aeruginosa* when adhering to each of these materials was then evaluated by means of global proteomic analysis relative to the physicochemical properties of these materials.

## Materials and methods

### Bacterial strain and medium

*P. aeruginosa* PAO1 strain was used during this study. Frozen bacterial stocks of the strain were sub-cultured three times in Luria-Bertani broth (LB) and then adjusted to pH 7.4 at 37°C with vigorous orbital shaking (180 rpm).

### Materials

AISI 316 stainless steel (SS) (Goodfellow, Huntingdon, United Kingdom), borosilicate glass Petri dishes (G) (Schott, Mainz, Germany), and polystyrene Petri dishes (PS) (Gosselin, Borre, France) were used as the model surfaces. Before each experiment, these surfaces were cleaned as previously described (Kamgang et al., 2007). Briefly, all surfaces were soaked for 15 min in a 2% (v/v) solution of commercial RBS 35 detergent (Société des Traitements Chimiques de Surface, Lambersart, France), then rinsed five times for 5 min in sterile deionized water at 50°C and then a further five times for 5 min with sterile deionized water at room temperature (RT). The surfaces were stored for 24 h at RT in sterile deionized water to prevent any atmospheric carbon pollution before the experiment.

### Physicochemical characterization of the materials

The homogeneity of surface hydrophobicity was investigated by water contact angle measurements using the sessile drop technique (DSA100M goniometer, Krüss, Palaiseau, France). This apparatus deposited a sterile deionized water droplet (300 pL) with a piezo dosing unit every 1 mm on an area of 10 × 20 mm^2^. The droplets were monitored for 2 s using a fast CCD (Charge Coupled Device) camera with 4x zoom and a 20x microscope objective. An automatic procedure was used for drop generation, image acquisition, contact angle determination, and the mapping of sample hydrophobicity. For each material, a cartography of wettability was constructed using Matlab 2011b software (Mathworks, Natick, USA) with a color map of visible colors from blue to red representing the contact angle to water (θ) from 0 to 100°. The average contact angle was determined from the 231 drops. All measurements were performed in triplicate.

Surface roughness was investigated as previously described (Poncin-Epaillard et al., 2013) using a surface profilometer (Perthometer M2, Mahr, Igny, France). The R_a_ (arithmetic average roughness) and R_max_ (maximum peak to valley height) parameters were calculated from the altitude of a stylus on seven consecutive sections of 0.8 mm. The experiments were conducted six times on each sample.

### Adhesion assays

Adhesion assays were performed in 150 mM NaCl to prevent bacterial growth and a shift to a biofilm phenotype. The viability of *P. aeruginosa* in this medium was tested and confirmed for an extended time of 72 h. *P. aeruginosa* PAO1 cultures grown overnight were washed three times in NaCl 150 mM and their concentrations were adjusted to 10^8^ CFU/mL (Colony Forming Unit per mL). The three model surfaces were covered with this bacterial suspension (to a depth of 0.5 cm) for 3 h at 37°C. Each material was then rinsed six times with 150 mM NaCl to remove any non- or weakly-attached bacteria. For direct observations, the fluorescent labeling of bacteria was achieved by immersing each contaminated material in distilled water containing SYTO9 1 μM (Molecular Probes, Life Technologies, Saint Aubin, France). Epifluorescence microscopic observations of the materials (Leica Microsystems, Nanterre, France) were performed using a 20X objective. The coverage rate of the bacteria adhering to the materials was determined using ImageJ (Rasband, 1997; Schneider et al., 2012). To enumerate the adherent cells, harvesting from the surfaces was performed with a cell scraper (Sarstedt, Newton, USA) in 150 mM NaCl. The cells were then centrifuged (10,000 g, 30 min, 4°C) and the pellets were stored at –80°C. Enumeration on LB agar plates enabled a determination of the number of bacteria that had thus been detached from each material.

### Total protein extraction

Bacterial pellets (10^9^ CFU) were lysed with 20 μL of 10% sodium dodecyl sulfate (SDS, Sigma-Aldrich, Lyon, France), sonicated three times for 5 min at 4°C and then centrifuged (10,000 g, 10 min, 4°C). Supernatants containing soluble proteins were recovered and mixed with an equivalent volume of Laemmli 2X running buffer (Biorad, Marnes-la-Coquette, France).

### Protein digestion

The proteins thus extracted were reduced with 5 mM dithiothreitol (Sigma-Aldrich), and alkylated with 25 mM iodoacetamide for 45 min in the dark. The protein sample was mixed with SDS loading buffer (63 mM Tris–HCl, pH 6.8, 10 mM DTT, 2% SDS, 0.02% bromophenol blue, 10% glycerol), then loaded onto a SDS-PAGE stacking gel (7%) and subjected to a short period of electrophoresis (10 mA, 50 V, 5 min). After migration, the gels were stained with Coomassie blue and de-stained with a solution containing 50% ethanol, 10% acetic acid, and 40% deionized water. The protein band revealed was then excised, washed with water, and subjected to protein digestion with 300 ng trypsin (Promega France, Charbonnières, France) in 25 mM ammonium bicarbonate buffer at 37°C. After overnight hydrolysis, tryptic peptides were recovered by extraction (Shevchenko et al., 2006), dried in under a speed vacuum and finally suspended in 25 μL nano-LC loading buffer (0.08% trifluoroacetic acid, 2% acetonitrile; Sigma-Aldrich). The peptides were then dried and stored at −20°C.

### Tandem mass spectrometry

Peptide analyses were performed on an Ultimate 3000 RSLC Nanosystem (Dionex, Voisins le Bretonneux, France) coupled to an LTQ-Orbitrap Discovery mass spectrometer (Thermo Fisher, Waltham, USA; PAPPSO proteomic platform, INRA, Jouy-en-Josas) with a nanoelectrospray interface. Briefly, 4 μL of tryptic digest were loaded onto a Pepmap100C18 pre-column (0.3 mm ID x 5 mm, 5 μm, Dionex) at a flow rate of 20 μL/min for 4 min. All peptides were separated onto a Pepmap100C18 column (0.075 mm ID × 500 mm, 2 μm, Dionex) with a gradient from 2 to 35% for 68 min at 300 nL/min and 40°C, for a total running time of 85 min which included the regeneration and equilibration steps of the column. Ionization was performed in positive mode (1.4 kV ionization potential) with a liquid junction and capillary probe (PicoTip Emiter, 10 μm ID; New Objective, Woburn, MA, USA). Data were acquired using Xcalibur (v2.07, Thermo Fisher Scientific) using a data-dependent method comprising two steps: first, a full scan MS in an Orbitrap analyser (from 300 to 1,400 in profile mode with a resolution of 15,000 at *m/z* 400) and second, fragmentation and the detection of daughter ions in the linear trap (*qz* = 0.22, activation time 50 ms and collision energy fixed at 35%) in centroid mode. The dynamic exclusion time was set at 45 s. To enhance mass accuracy, the lock mass option was activated on dimethylcyclosiloxan (*m/z* 445.120029).

### Protein quantification

The raw files produced under Xcalibur were first converted to mzXML files with MSconvert (http://sashimi.sourceforge.net, version 3.0.3768) and in a second step, proteins were identified using X!tandem software (X!tandem Piledriver 2015.04.01.1; http://www.thegpm.org) (Craig and Beavis, 2004) against a *P. aeruginosa* PAO1 protein database (NCBInr, NC_002516.2; 5566 sequences) associated with a standard proteomic contaminant database. The X!Tandem search parameters were (i) trypsin specificity with three missed cleavages and (ii) variable oxidation states of methionine. Semi-tryptic peptide detection was included by activating the option in the data refine mode. The mass tolerance was fixed at 10 ppm for precursor ions and 0.5 Da for fragment ions. The final search results were filtered using a multiple threshold filter applied at the protein level and consisting of a Log10 protein E-value lower than −2.6 identified with a minimum of two different peptide sequences, detected in at least one analysis as having a peptide E-value lower than 0.05. These criteria led to false discovery rates estimated using the decoy database of 0.07 and 0.10% for peptide and protein identification, respectively.

X!TandemPipeline 3.3.5 analysis (Langella et al., 2017) (http://pappso.inra.fr/bioinfo/xtandempipeline/) was used to filter and group the peptide/protein identifications from MS/MS mass spectra.

The mass spectrometry proteomics data have been deposited to the ProteomeXchange Consortium *via* the PRIDE (Vizcaíno et al., 2016) partner repository with the dataset identifier PXD006881 and 10.6019/PXD006881. PRIDE Converter 2 was used to convert our data in PRIDE-xml format (Côté et al., 2012). The submission was achieved using ProteomeXchange (Vizcaíno et al., 2014).

The mass spectrometry proteomics data have been also deposited on the PROTICdb platform (http://moulon.inra.fr/protic/p_aeruginosa_adhesion).

### Statistical analyses

Physicochemical results (contact angles and roughness) were considered to be significantly variable using ANOVA when the *p-value* was <0.05.

A spectral counting approach was used to identify proteins detected in statistically different quantities between the conditions. Data from the mass spectrometry protein quantification of five biological replicates were compared. First, all conditions were compared (70 proteins significantly different) and second, the conditions were compared between each other and the results were merged.

Filtering and statistical analysis were performed using MassChroqR, an R package developed by PAPPSO (http://pappso.inra.fr/). A small difference in the number of spectra may be noise-induced, so proteins displaying a minor variation between the conditions (whose mean difference was less than three spectra) were discarded from the analysis. A generalized linear mixed model (GLM) with a Poisson distribution was applied. This model is appropriate in the case of a counting such as spectral counting. The significance of protein abundance changes was determined by analysis of variance ANOVA using a Chi-square test. A protein was considered to be significantly variable when the *p-value* was <0.01. The *p-values* were adjusted for multiple comparisons using Benjamini and Hochberg's method (Benjamini and Hochberg, 1995).

### Heat map representation

Heat maps were generated under MassChroqR using the heatplot function from the made4 package (http://www.bioconductor.org). The heat map revealed a gradient of colors assigned to the different numbers of spectra (centered and reduced) and the dendrogram provided a graphical representation of a hierarchical cluster with distances calculated using the Pearson correlation coefficient (default distfun argument of the heatplot function of the “made4” of the R package); the aggregation criterion was the average-linkage or UPGMA (Unweighted Pair Group Method with Arithmetic mean).

### PseudoCAP functional classification and GO (gene ontology) term annotations

Functional classifications were performed using the facilitating community-based PseudoCAP (*Pseudomonas Community Project*) functional classes (http://www.pseudomonas.com) (Winsor et al., 2016). GO term annotations based on mappings to InterPro functional domain predictions provide additional information.

## Results

### Physicochemical properties of the surfaces of different materials

**Figure 1:**
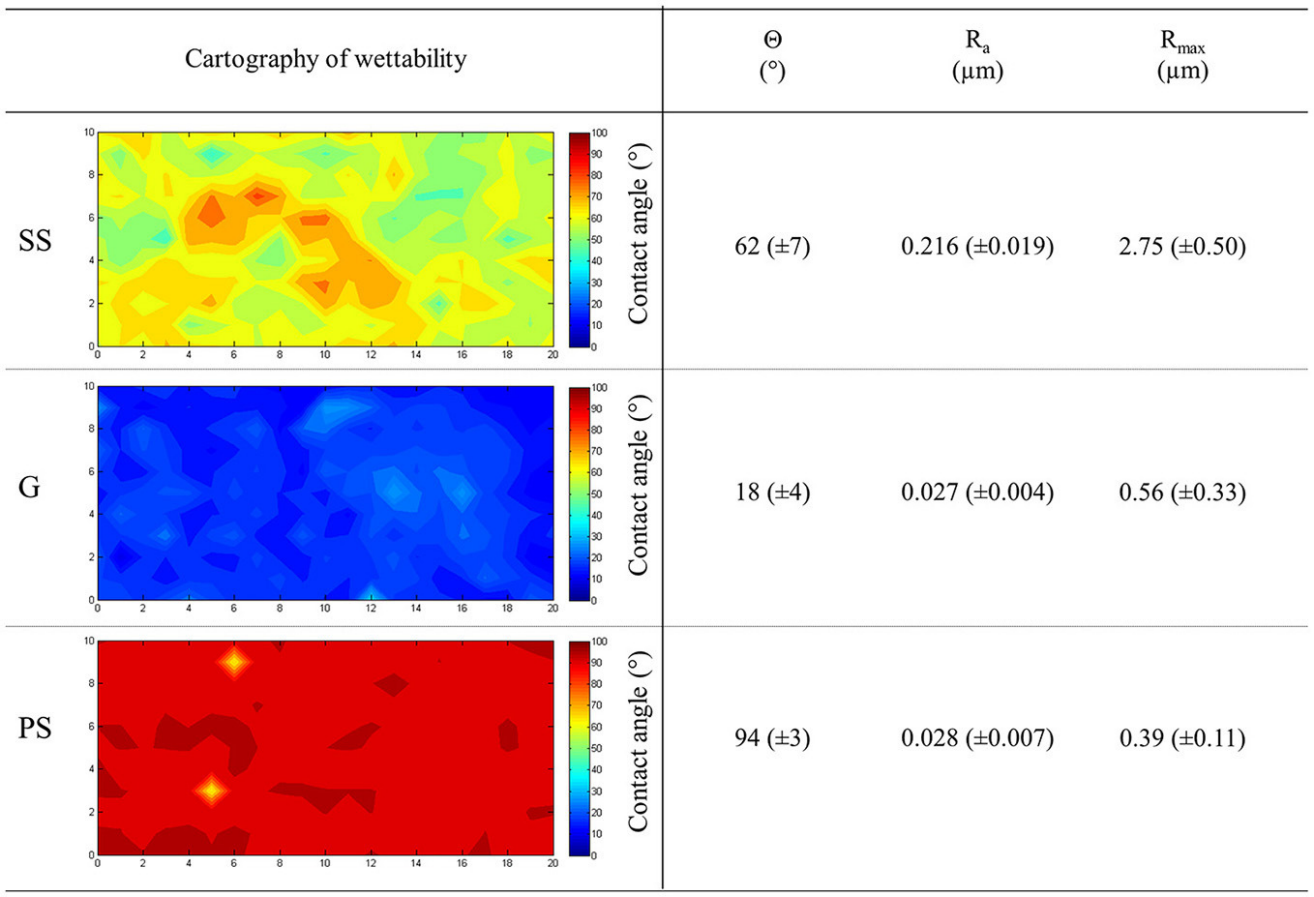
Physicochemical properties of the materials. Stainless steel (SS), glass (G), and polystyrene (PS) were assayed for their wettability. The average contact angles (Θ) and the roughness parameters R_a_ and R_max_ were also reported. Standard deviations are given into brackets.

Surface hydrophobicity or hydrophilicity can be characterized by water surface wettability, measured using contact angles (θ) (Boulangé-Petermann et al., 1997). A cartography of wettability was constructed by measuring 231 contact angles per material. As shown in Figure 1, stainless steel (SS), glass (G), and polystyrene (PS) displayed homogeneous wettability which ensured the homogeneous adhesion of bacteria. Under our experimental conditions, SS was considered to be moderately hydrophobic (62° ± 7°), G as hydrophilic (18° ± 4°), and PS as hydrophobic (94° ± 3°) (Figure 1), providing an hydrophobic range for adhesion studies with materials exhibiting statistically different contact angles (*p* < 0.05). Concerning the topography of these surfaces, SS was shown to be rough, with an R_a_ of 0.216 μm, while G and PS were considered as smoother surfaces with R_a_ = 0.027 and 0.028 μm, respectively (Figure 1). The R_max_ parameter (maximum peak to valley height) was clearly higher for SS than for G and PS, suggesting the presence of crevices on the SS surface (Boulangé-Petermann et al., 1997). SS roughness (R_a_ and R_max_) was shown to differ statistically from that of G and PS, while the roughness of G and PS was statistically similar (*p* < 0.05). Taken together, our data led us to conclude that the three studied surfaces presented different physicochemical properties, i.e., SS was moderately hydrophilic and rough, potentially containing crevices, G was hydrophilic and smooth while PS was hydrophobic and smooth. As the number of adherent bacteria could be related to the physicochemical properties of materials (Bellon-Fontaine et al., 1990), the adhesion of *P. aeruginosa* was investigated on these different surfaces.

### Materials impact the adhesion capability of *P. aeruginosa*

**Figure 2:**
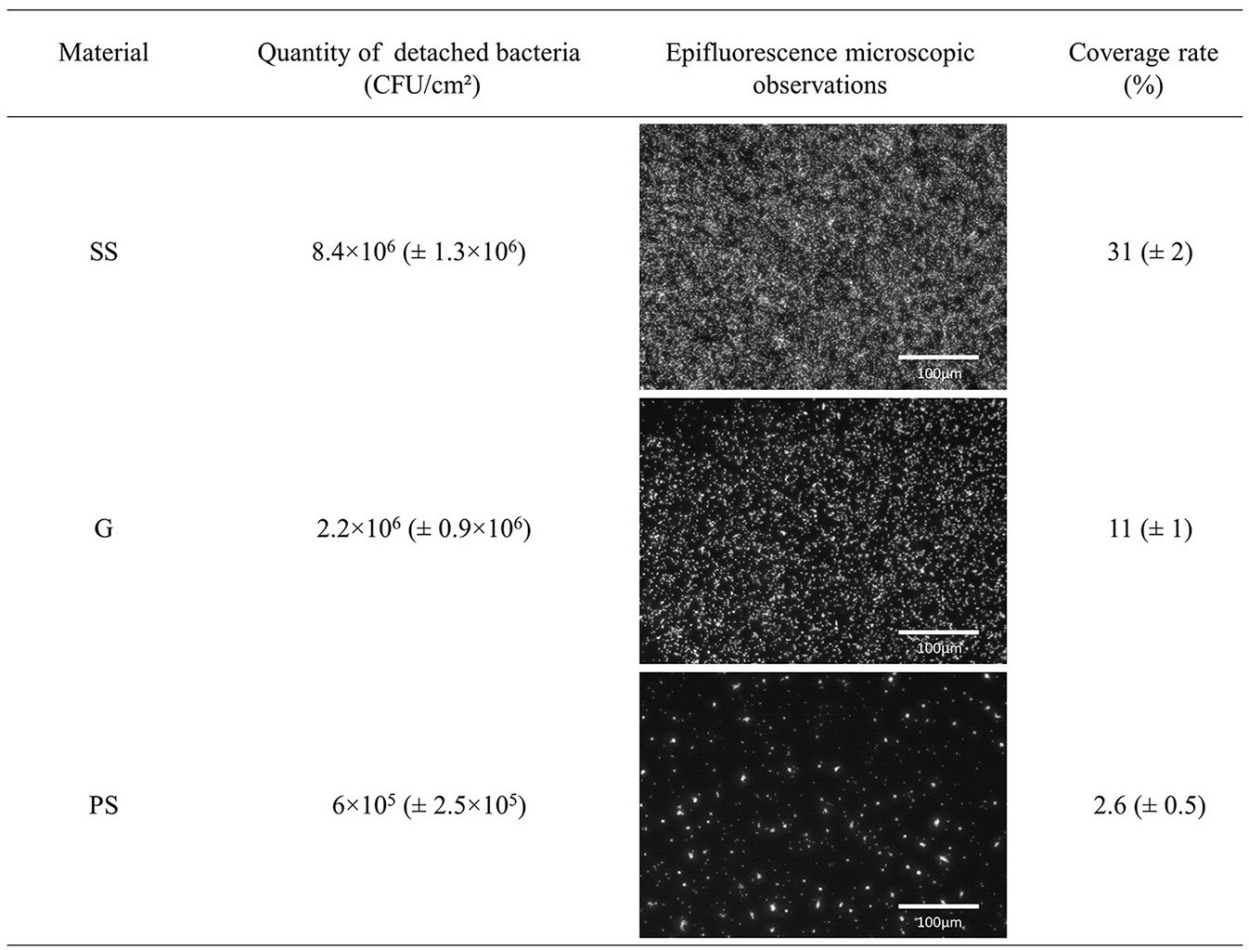
Adhesion of *P. aeruginosa* PAO1 to stainless steel (SS), glass (G), and polystyrene (PS), 3 h at 37°C. Quantity of adherent *P. aeruginosa* PAO1 determined by enumeration and epifluorescence microscopic observations with a ×20 objective after SYTO9 staining.

Bacteria were allowed to adhere to these three surfaces for 3 h before being recovered and observed or counted. Figure 2 shows that 8.4 × 10^6^ (± 1.3 × 10^6^) CFU/cm^2^, 2.2 × 10^6^ (± 0.9 × 10^6^) CFU/cm^2^, and 6 × 10^5^ (± 2.5 × 10^5^) CFU/cm^2^ (*P* < 0.05) were detached from SS, G, and PS, respectively, indicating that the material had an impact on the number of adherent cells. Direct epifluorescence microscopic observations of the contaminated materials (Figure 2) were correlated to the enumeration results by showing a higher coverage rate of bacteria on SS (31%), an intermediate rate on G (11%), and a lower rate on PS (2.6%). Taken together, these data suggest that *P. aeruginosa* cells were more likely able to adhere to SS and G rather than PS surfaces under our experimental conditions.

### *P. aeruginosa* displays varied global proteome patterns in response to adhesion on different materials

**Figure 3:**
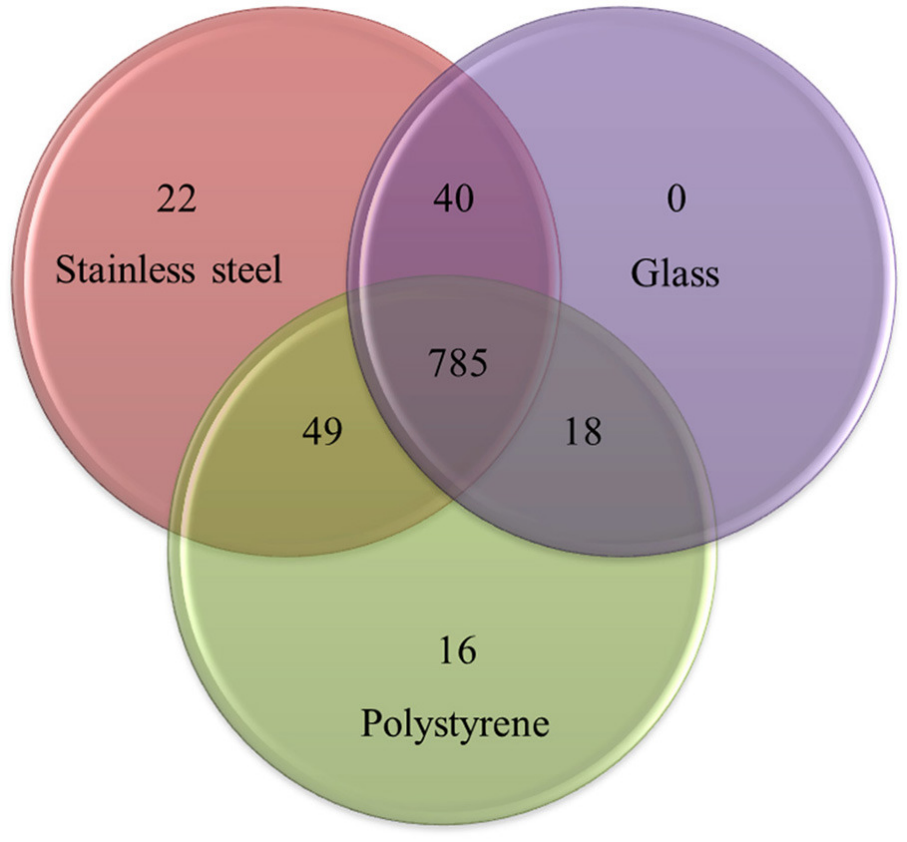
Venn diagram based comparisons indicating the number of total detected proteins (930) from *P. aeruginosa* adhering to stainless steel, glass, and polystyrene. The complete list of these proteins is available in Table S1.

**Figure 4:**
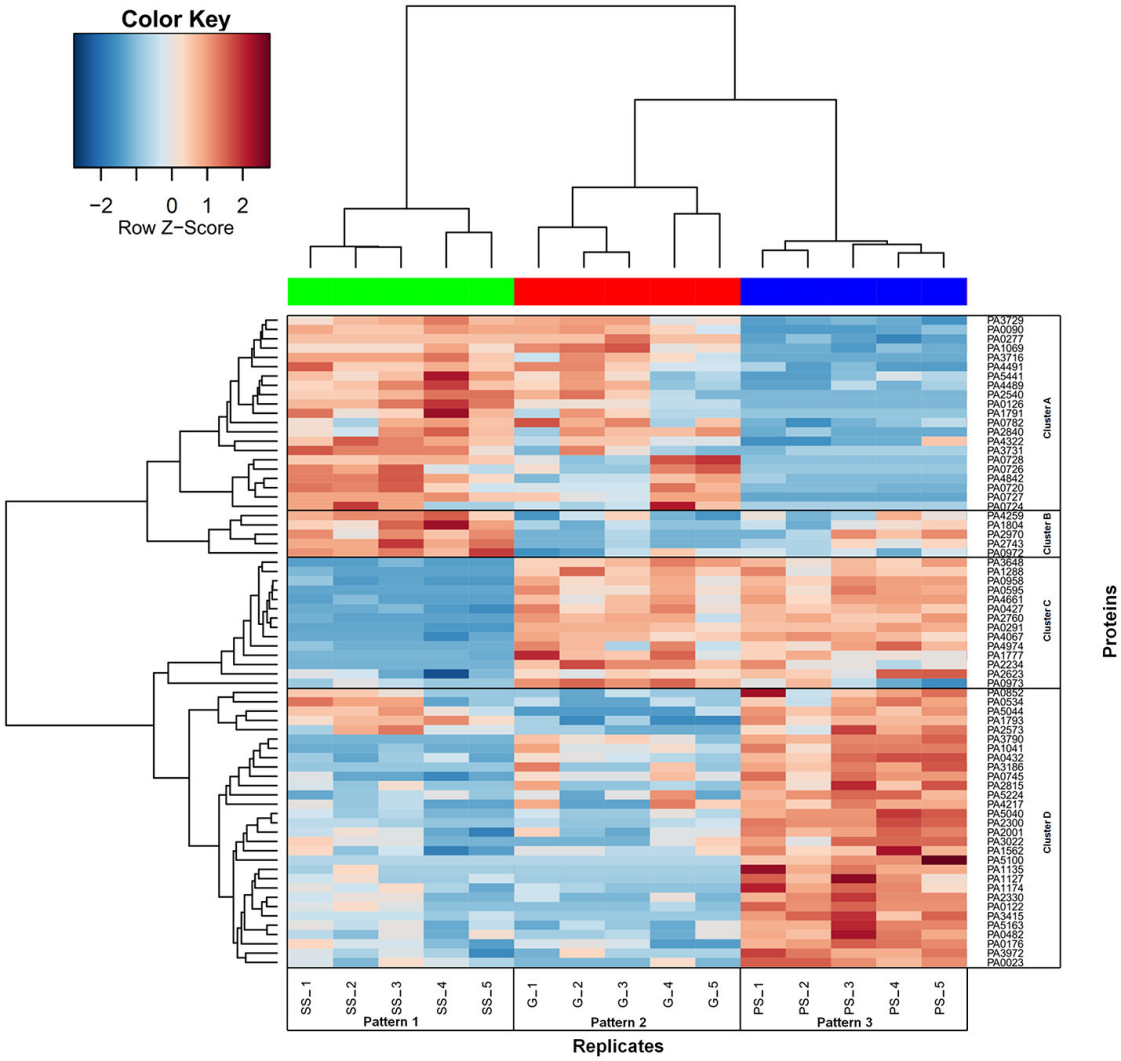
Normalized number of spectra of the 70 proteins with significant differences of quantities after adhesion of *P. aeruginosa* cells to stainless steel (SS), glass (G), and polystyrene (PS). Quantity of each protein is normalized from the number of spectra counted by mass spectrometry. The 70 proteins identified as significantly altered in abundance between SS, G, and PS were sorted by heatplot function. Replicates are indicated by numbers 1 to 5 and are referred to patterns 1 to 3 (1 corresponds to SS, 2 to G, and 3 to PS). Proteins are designated by their locus tag referenced in Table S4. Four clusters of proteins (A, B, C, and D) following the same abundance pattern are represented by black boxes.

The total proteome of *P. aeruginosa* PAO1 adhering to SS, G and PS was analyzed using nano-LC-MS/MS and X!TandemPipeline. The mass spectra thus generated (Table S1) enabled the identification of 930 proteins (Table S2) out of the 5,570 predicted ORFs that are encoded by the *P. aeruginosa* PAO1 genome. Of these, 785 were identified on the three materials, 107 on two of them, and 38 were detected specifically on only one surface (Figure 3, Tables S2, S3). Using strong statistical validation assays, the abundance of 70 out of these 930 proteins was shown to differ between the materials compared by pairs (Table S4). *P. aeruginosa* displayed differential abundance of the greatest number of proteins (or a differential abundance of peptide ions) when adhesion was compared between SS and PS (57 proteins). Among the 834 proteins identified on these two materials (Figure 3), 57 proteins showed significant differences in their abundances: four were specifically present in this pair comparison, 8 in SS vs. G and 7 in PS vs. G. By contrast, comparing SS vs. G and PS vs. G led to 21 and 39 differentially abundant proteins (Table S4). Taken together, these data showed a strong bacterial response that was specific to the material used for adhesion. The data were then subjected to hierarchical clustering which focused on the 70 proteins that displayed differential content following adhesion on at least two materials. The abundance of these 70 proteins was compared using the normalized quantities of spectral counting from five biological replicates (Figure 4, Table S4). Protein levels were represented as a color map ranging from red to blue, indicating proteins produced at lower and higher levels, respectively. Clustering of the five replicates highlighted a strong coherence for each material in terms of protein abundance, which demonstrated the reproducibility of our assays (Figure 4, upper dendrogram). Clustering of the 70 proteins in terms of their abundance on a given material, followed by a comparison of these results on the three materials, enabled the identification of a further four clusters (Figure 4, left dendrogram, clusters A, B, C, and D). Under these conditions, each cluster enabled the grouping of proteins with a relatively similar level on a particular material compared to the two others. SS-adherent bacteria presented more proteins belonging to the A- and B- clusters, while G-adherent bacteria displayed more proteins from the A- and C- clusters. PS-adherent bacteria were characterized by an abundance of proteins belonging to the C- and D-clusters. Taken together, this clustering led to the definition of patterns on each material which reflected protein abundance profiles considering A to D clusters on a given material, vs. the other two (Figure 4). Remarkably, this global approach permitted the identification of three protein abundance patterns that were each relevant to a specific material.

### Physiological response to the materials of adherent *P. aeruginosa*

To obtain further insights into the physiological response of *P. aeruginosa* to adhesion on each of the three materials, the functional classes of the 70 proteins whose abundance differed significantly between at least two materials were assigned according to the PseudoCAP classification (Winsor et al., 2016). The functional classes relative to all differentially abundant proteins are available in Table 1 and Table S5.

**Table 1:** PseudoCAP-based functional classes of the significantly differentially abundant proteins between the materials

				**Average**^b^			***p adj***^c^			**Cluster**^d^			
**PseudoCAP-based functional classes^a^**	**Protein name**	**Locus tag**	**Gene name**	**SS**	**G**	**PS**	**SS/G**	**SS/PS**	**PS/G**	**A**	**B**	**C**	**D**
**Adaptation, Protection**	RahU	PA0122	*rahU*	1.4	0.2	5.4		8.9E-04	3.1E-07				
	LPS-assembly protein LptD	PA0595	*lptD*	0.0	11.0	13.0	1.5E-16	8.1E-20					
	MagD	PA4489	*magD*	11.6	7.8	2.6		1.3E-07	6.0E-04				
	probable oxidoreductase	PA1127		0.8	0.0	4.2		8.5E-04	4.5E-07				
**Chemotaxis**	probable chemotaxis transducer	PA2573		2.8	0.4	4.0			1.1E-04				
	aerotaxis transducer Aer2	PA0176	*aer2*	6.6	5.8	15.0		1.2E-04	1.7E-05				
**Amino acid biosynthesis and metabolism**	S-adenosyl-L-homocysteine hydrolase	PA0432	*sahH*	9.6	12.0	18.0		7.4E-04					
	proline dehydrogenase PutA	PA0782	*putA*	17.0	18.6	10.4		8.6E-03	1.6E-03				
**Central intermediary metabolism**	malate synthase G	PA0482	*glcB*	13.0	12.4	20.4		7.7E-03	3.5E-03				
**Carbon compound catabolism**	chitinase	PA2300	*chiC*	1.0	1.4	11.2		1.7E-11	6.5E-10				
**Energy metabolism**	periplasmic nitrate reductase protein NapA	PA1174	*napA*	2.8	1.6	8.0		7.3E-04	7.7E-06				
	aconitate hydratase 1	PA1562	*acnA*	12.6	15.6	21.2		1.9E-03					
	urocanate hydratase	PA5100	*hutU*	0.0	0.0	3.2		8.6E-06	1.1E-05				
	probable dihydrolipoamide acetyltransferase	PA3415		0.8	0.0	4.6		3.0E-04	1.5E-07				
	quinone oxidoreductase	PA0023	*qor*	1.6	0.6	5.2		3.2E-03	1.7E-05				
	isocitrate dehydrogenase	PA2623	*icd*	22.2	28.8	32.0		5.6E-03					
**Fatty acid and phospholipid metabolism**	acetyl-CoA acetyltransferase	PA2001	*atoB*	4.0	5.0	10.6		2.2E-04	3.0E-03				
**DNA replication, recombination, modification and repair**	DNA-binding protein HU	PA1804	*hupB*	12.4	5.4	7.6	5.3E-04						
**Transcription, RNA processing and degradation**	probable ATP-dependent RNA helicase	PA2840		4.0	3.8	0.2		1.4E-05	2.8E-05				
**Translation, post-translational modification, degradation**	aminopeptidase P	PA5224	*pepP*	1.4	2.4	6.0		2.2E-04	8.6E-03				
	50S ribosomal protein L32	PA2970	*rpmF*	3.4	0.2	2.4	1.2E-04						
	30S ribosomal protein S19	PA4259	*rpsS*	8.6	3.8	5.4	5.7E-03						
	translation initiation factor IF-3	PA2743	*infC*	5.6	1.2	3.0	2.8E-04						
	peptidyl-prolyl cis-trans isomerase B	PA1793	*ppiB*	4.8	0.8	5.0	2.4E-04		1.1E-04				
**Transport of small molecules**	OprQ	PA2760	*oprQ*	0.2	10.6	11.2	1.7E-14	1.6E-15					
	glucose/carbohydrate outer membrane porin OprB precursor	PA3186	*oprB*	0.0	2.0	5.4		5.4E-09	8.1E-03				
	basic amino acid, basic peptide and imipenem outer membrane porin OprD precursor	PA0958	*oprD*	1.0	13.6	16.0	1.7E-14	1.1E-17					
	putative copper transport outer membrane porin OprC precursor	PA3790	*oprC*	0.0	7.4	14.4	7.8E-12	1.8E-21	1.7E-03				
	TolB protein	PA0972	*tolB*	15.4	6.0	6.6	1.7E-05	6.3E-05					
	anaerobically-induced outer membrane porin OprE precursor	PA0291	*oprE*	0.6	11.2	11.6	3.7E-13	8.2E-14					
	probable outer membrane protein precursor	PA1288		0.2	8.2	7.4	2.4E-11	3.6E-10					
	outer membrane protein Opr86	PA3648	*opr86*	0.2	7.2	6.6	5.7E-10	4.2E-09					
	probable outer membrane protein precursor	PA1041		0.2	3.6	6.4	6.7E-05	6.8E-09					
	peptidoglycan associated lipoprotein OprL precursor	PA0973	*oprL*	2.6	7.2	2.8	2.4E-03		3.3E-03				
**Membrane proteins**	lipid A 3-O-deacylase	PA4661	*pagL*	1.2	6.4	7.6	4.2E-05	1.4E-06					
	outer membrane protein OprG precursor	PA4067	*oprG*	1.6	10.6	11.4	6.4E-09	8.0E-10					
	major intrinsic multiple antibiotic resistance efflux outer membrane protein OprM precursor	PA0427	*oprM*	1.6	12.2	11.8	8.1E-11	3.2E-10					
	major porin and structural outer membrane porin OprF precursor	PA1777	*oprF*	7.8	26.6	20.6	2.0E-12	2.0E-07					
**Cell wall/LPS/capsule**	PslD	PA2234	*pslD*	0.0	3.8	2.2	1.4E-06						
	glucose-1-phosphate thymidylyltransferase	PA5163	*rmlA*	5.0	4.2	9.0			5.6E-03				
**Motility and attachment**	type 4 fimbrial biogenesis outer membrane protein PilQ precursor	PA5040	*pilQ*	1.2	2.2	8.8		5.5E-08	1.7E-05				
	type 4 fimbrial biogenesis protein PilM	PA5044	*pilM*	5.6	2.4	6.6			3.1E-03				
**Protein secretion/export apparatus**	probable outer membrane protein precursor	PA4974		0.2	6.0	7.2	2.8E-08	6.7E-10					
	ClpV1	PA0090	*clpV1*	13.4	12.0	0.8		2.6E-15	5.6E-13				
**Related to phages, transposon or plasmid**	probable coat protein A of bacteriophage Pf1	PA0724	*coaA*	3.6	3.0	0.0		2.2E-06					
	hypothetical protein of bacteriophage Pf1	PA0726		4.2	3.2	0.0		3.0E-07	1.1E-05				
	hypothetical protein from bacteriophage Pf1	PA0727		10.8	8.4	0.0		1.1E-16	5.6E-13				
	helix destabilizing protein of bacteriophage Pf1	PA0720		16.8	11.8	0.6		1.3E-20	2.6E-13				
	probable bacteriophage integrase	PA0728		5.4	4.6	0.0		5.4E-09					
**Secreted factors (toxins, enzymes, alginate)**	chitin-binding protein CbpD precursor	PA0852	*cbpD*	2.6	1.0	5.0			3.8E-04				
**Putative enzymes**	FAD-dependent oxidoreductase	PA0534	*pauB1*	3.4	1.0	4.4			1.7E-03				
	probable acyl-CoA dehydrogenase	PA2815		3.0	3.2	7.4		3.9E-03	6.7E-03				
	probable acyl-CoA dehydrogenase	PA3972		2.0	2.2	5.8		3.8E-03	7.1E-03				
	probable enoyl-CoA hydratase/isomerase	PA0745		2.4	5.6	8.0		1.8E-04					
	flavin-containing monooxygenase	PA4217	*phzS*	1.2	3.0	5.0		8.9E-04					
**Hypothetical proteins**	hypothetical protein	PA2330		1.6	1.0	6.2		3.5E-04	1.7E-05				
	conserved hypothetical protein	PA1135		0.4	0.0	3.8		1.8E-04	1.7E-06				
	hypothetical protein	PA3022		1.4	1.0	4.2			2.6E-03				
	hypothetical protein	PA0126		3.8	1.6	0.0		1.2E-06					
	conserved hypothetical protein	PA2540		6.4	4.2	0.0		2.7E-10	4.5E-07				
	hypothetical protein	PA1791		5.8	2.6	0.0		1.5E-09					
	conserved hypothetical protein	PA3731		7.6	4.0	2.8		1.5E-03					
	hypothetical protein	PA5441		5.8	3.4	1.2		1.4E-04					
	hypothetical protein	PA3716		10.0	7.0	0.0		1.5E-15	5.9E-11				
	conserved hypothetical protein	PA3729		22.4	21.4	9.6		1.2E-06	8.2E-06				
	hypothetical protein	PA1069		8.6	10.8	2.0		8.6E-06	9.7E-08				
	conserved hypothetical protein	PA0277		6.0	6.4	1.0		2.7E-05	1.2E-05				
	conserved hypothetical protein	PA4322		7.2	4.6	1.6		3.5E-05					
	hypothetical protein	PA4842		6.4	3.0	0.2		6.8E-09					
	MagB	PA4491	*magB*	5.8	5.0	0.6		2.7E-06	2.8E-05				

a *PseudoCAP-based functional classes of the proteins (p-adjust < 0.01) significantly differentially abundant showing a maximal variation of three spectra between the replicates of a same material*.

b *average of peptide spectra number of the five replicates for SS, G and PS*.

c *p adj values from ANOVA analyses were reported only for significant results (p < 0.01). SS was compared to G (SS/G) and PS (SS/PS). PS was compared to G (PS/G). Shaded blue p adj values corresponded to under-production of the protein on SS compared to PS or G and PS compared to G. Conversely, shaded red p adj values corresponded to over-production of SS compared to PS or G and PS compared to G*.

d *Clusters A, B, C, and D determined from heatplot function (Figure 4). Each cluster allows the grouping of proteins with a relative similar abundance tendency on a given material, compared to the two other materials*.

#### Proteomic response of *P. aeruginosa* adhering to SS compared to G (SS/G)

Based on PseudoCAP analysis, most proteins belonged to three functional classes (Table 1). These included the “Membrane proteins” and “Transport of small molecules” classes, in which the great majority of the affected proteins were less abundant when *P. aeruginosa* was allowed to adhere to SS/G. Conversely, more abundant proteins were mostly found in the “Translation, post-translational modification, degradation” class. Numerous outer membrane proteins (OMPs) were produced at markedly lower levels when the bacteria were allowed to adhere to SS/G (Table 1). This was notably the case for many major porins, including the non-specific major porin OprF, the specific channels OprD, OprE, OprQ, OprC, OprG, and the OprM OM component of the MexAB/OprM efflux pump. It was also the case for Opr86, which is involved in porin folding into the OM (Tashiro et al., 2008), and for the peptidoglycan associated lipoprotein (Pal) OprL, a member of the Tol-Pal complex that is important to maintaining OM integrity in Gram negative bacteria (Lazzaroni et al., 1999). In addition, lipid A deacylase (PagL), which is involved in lipopolysaccharide (LPS) modification, was also less abundant when *P. aeruginosa* was allowed to adhere to SS/G. Conversely, four proteins belonging to the “Translation” PseudoCAP functional class, such as the ribosomal proteins RpmF, RpsS, the translation initiation factor InfC, the peptidyl-prolyl cis-trans isomerase PpiB and the TolB protein belonging to the “Transport of small molecules” class, were found to be produced at higher levels when bacteria were allowed to adhere to SS/G.

#### Proteomic response of *P. aeruginosa* adhering to SS compared to PS (SS/PS)

Based on PseudoCAP analysis, most of the dysregulated proteins belonged to eight functional classes (Table 1). These included the “Transport of small molecules,” “Membrane proteins,” “Putative enzymes,” “Energy metabolism,” and “Adaptation, protection” classes, in which the great majority of affected proteins were less abundant when *P. aeruginosa* was allowed to adhere to SS/PS. By contrast, proteins included in the “Hypothetical proteins” and “Related to phages, transposon or plasmid” functional classes were found at higher abundance after the adhesion of *P. aeruginosa* to SS/PS (Table 1, Table S5). Numerous OMPs were less abundant when *P. aeruginosa* was allowed to adhere to SS/PS. Interestingly, many of them, including the previously described porins, the porin folding chaperone Opr86 and the LPS lipase PagL, were also found at lower levels when SS and G were compared. Remarkably, and as indicated elsewhere regarding the abundance of the 70 proteins that were differentially produced between the three materials, this led to grouping all of these OMPs in a single cluster (Figure 4, cluster C). In addition, the glucose-specific porin OprB, the OMP precursors PA1041, PA1288, and PA4974, the LPS-assembly protein LptD and the type 4 fimbrial biogenesis OMP PilQ (which is involved in the production of adhesive organelles type IV pili) were also produced less when bacteria were allowed to adhere to SS/PS. In addition, 10 proteins in the energy, amino acid, carbon compound, central, or fatty acid metabolism pathways were down-regulated when *P. aeruginosa* was allowed to adhere to SS/PS. For example, this was the case for the periplasmic nitrate reductase protein NapA, urocanate hydratase, aconitate hydratase 1, probable dihydrolipoamide acetyltransferase, quinone oxidoreductase and isocitrate dehydrogenase which are involved in energy metabolism. In addition, 3 proteins belonging to the “putative enzymes” class, PA2815, PA3972, and PA0745, were less abundant in this condition and might be involved in lipid homeostasis (Table S5). Conversely, several proteins were more abundant in the context of SS/PS. This was the case for PA0720, PA0724, PA0726, PA0727, and PA0728, which are components of the Pf1 bacteriophage. PA0724, PA0726, PA0727, and PA0728 were not detected on PS and PA0720 was about 30 times more abundant when the bacteria were allowed to adhere to SS/PS. Similarly, the type 6 secretion system ATPase ClpV1, and the Tol-Pal periplasmic TolB component that is involved in cell wall integrity maintenance, were found to be more abundant when the bacteria adhered to SS/PS. Finally, the ATP-dependent RNA helicase that is involved in transcription was more abundant on SS/PS. Interestingly, most of the hypothetical proteins were more abundant in this condition and for several their predicted activity is related to the envelope metabolism, i.e., PA2540 is a putative phospholipase, and PA1791, PA4842, and PA4491 are putative membrane proteins.

#### Proteome response of *P. aeruginosa* adhering to PS compared to G (PS/G)

Based on PseudoCAP analysis, most of the dysregulated proteins belonged to six functional classes (Table 1). These included the “Energy metabolism,” “Adaptation, protection,” “Putative enzymes,” and “Motility and attachment” classes, where the great majority of affected proteins were up-regulated when *P. aeruginosa* was allowed to adhere to PS/G. By contrast, down-regulated proteins were mostly found in the “Hypothetical proteins” and “Related to phages” classes. Several metabolic proteins were over-produced on PS/G, namely periplasmic nitrate reductase NapA, urocanate hydratase, dihydrolipoamide acetyltransferase, and quinone oxidoreductase, all involved in energy metabolism and malate synthase G related to central intermediary metabolism. Chitinase that is involved in carbon catabolism is regulated by quorum sensing (QS) in *P. aeruginosa*. The over-production of ChiC in response to adhesion on PS might thus reflect some activity of QS. Similarly, two proteins involved in chemotaxis (PA2573 and Aer2), and two proteins related to pili biogenesis (PilQ and PilM) were over-produced on PS/G. Other proteins were found in smaller quantities on PS/G, including MagB which is potentially involved in host-pathogen interactions (Robert-Genthon et al., 2013), and the three phage-related proteins PA0720, PA0726, and PA0727.

Taken together, these data showed that the abundance of numerous proteins was modified as a function of the material to which bacteria adhered, enabling the identification of abundance patterns that appeared to be specific to a given surface.

## Discussion

This study was designed to use a proteomic approach to highlight the physiological responses of *P. aeruginosa* regarding its adhesion to three different surfaces that are commonly found in many environments, including hospitals and industry. It had previously been shown that bacterial adhesion to solid surfaces is dependent on a number of factors, e.g., physicochemical and mechanical interactions (Bos et al., 1999). Therefore, under our experimental conditions (static adhesion, high ionic strength), the number of adherent bacteria would mainly depend on the Lifshitz-van der Waals, Lewis acid-base and roughness characteristics of the materials, or in another words their hydrophobic/hydrophilic properties (Bruzaud et al., 2015). First of all, the three studied surfaces were shown to present different physicochemical properties: SS was moderately hydrophilic and rough, containing possible crevices, G was hydrophilic and smooth and PS was hydrophobic and smooth. Enumerations combined with microscopic assays revealed that *P. aeruginosa* PAO1, previously characterized in terms of its hydrophilic and electron-donor properties (Bruzaud et al., 2015), was able to adhere about 14- and 4-fold more to SS and G than to PS. This phenotype could at least partly explain the weaker interactions between *P. aeruginosa* and the hydrophobic material. Although SS was less hydrophilic than G, maximum adhesion was observed using SS. These observations may have resulted from the rougher surface of SS, potentially trapping the cells (Faille et al., 2000).

Given these results, which indicate different adhesion phenotypes as a function of the material involved, proteomic studies were performed to determine bacterial physiology in response to adhesion to these three materials. One technical problem was to recover sufficient adhered bacteria to be able to perform a proteomic study (at least 10^9^ CFU). Indeed, whereas the concentration of cells recovered from biofilms is clearly sufficient (Walker et al., 2005), under our conditions the concentration of recovered adherent bacteria was 8.4 × 10^6^ CFU/cm^2^ with SS, 2.2 × 10^6^ CFU/cm^2^ with G, and 6.0 × 10^5^ CFU/cm^2^ with PS. Large surface areas of the materials (120 cm^2^ for SS, 500 cm^2^ for G, and 1,700 cm^2^ for PS per replicate) were therefore used for the adhesion assays in order to recover 10^9^ CFU of bacteria. Furthermore, because this study was trying to determine the effects of the abiotic material on bacterial physiology, proper contact between the cells and surfaces was necessary to ensure that the majority of harvested cells had been affected. The adherent cells were observed using epifluorescence microscopy, which confirmed that most of the adherent cells were indeed in contact with the surface.

Total proteins were then extracted from *P. aeruginosa* that had been allowed to adhere to the three materials. Nano-LC MS/MS analyses enabled the identification of 930 proteins, representing about 16% of the open reading frames encoded by the *P. aeruginosa* genome. Among these, 70 proteins were identified as displaying a different abundance between at least two materials, representing about 7% of the total identified proteome. These observations were consistent with previous studies that had compared planktonic and biofilm cells and revealed similar quantities of dysregulated proteins (Vilain et al., 2004; Southey-Pillig et al., 2005). Remarkably, clustering of these proteins enabled the identification of three protein abundance patterns, each relevant to a specific material, suggestive of complex molecular mechanisms as a function of the relationships between cells and the surfaces of materials, as had previously been observed for bacteria adhering to agar surfaces of differing stiffness (Belas, 2014). The abundance of these 70 *P. aeruginosa* proteins varied according to the substrate, indicating that adhesion was probably complex and involved more than one protein (as shown by the three abundance patterns). A broad diversity of cellular functions was affected differently by the type of material, including mainly the “Membrane proteins,” “Transport of small molecules,” and “Metabolism” PseudoCAP functional classes. Taken together, these data clearly suggest that *P. aeruginosa* may develop a specific physiological state in response to such abiotic surfaces. To our knowledge, this is the first proteomic study to have shown such an impact of a substrate on the bacterial response, suggesting that *P. aeruginosa* is capable of the differential sensing of surfaces and modulates its physiology accordingly. This is the case for the sensing system responsible for type IV pili biogenesis (Whitchurch et al., 2004) that may be activated when the bacteria are grown on PS rather than other surfaces as shown with the increase on PS of two proteins belonging to type IV pili biogenesis pathway. This sensing system is involved in the surface-associated behaviors of *P. aeruginosa* as previously shown (Luo et al., 2015) but this role might be modulated by the nature of the surfaces.

We detected only one bacteriophage Pf1 protein with a very low level on PS when compared to SS and G. Although these proteins play a key role in host interactions and are largely involved in the formation of *P. aeruginosa* biofilms (Webb et al., 2004; Secor et al., 2015), their production seems not systematically enhanced when bacteria adhere to inert materials.

One of the most striking findings of this study was the variations in abundance of major porins and of porin folding chaperone Opr86, a member of the Omp85 family that is involved in OM biogenesis (Tashiro et al., 2008). Porins are proteins which form water-filled channels across the OM of Gram-negative bacteria and mediate the uptake or efflux of a number of compounds such as ions, small nutrient molecules, antibiotics, and large iron-siderophore complexes (Tamber et al., 2006). Porins are thus involved in controlling OM permeability and selectivity (Hancock and Farmer, 1993), and are also important actors in maintaining cell wall structure and homeostasis (Tashiro et al., 2008). OprF belongs to the OmpA family, which is a group of genetically related, surface-exposed porin proteins that are present in high-copy numbers in the OM of Gram-negative bacteria. OprF is a general porin that is non-covalently linked to the peptidoglycan layer, contributing thus to maintenance of the cell wall structure (Hancock and Carey, 1979). It is involved in adhesion, biofilm formation and virulence (Azghani et al., 2002; Sriramulu et al., 2005; Fito-Boncompte et al., 2011; Bouffartigues et al., 2015). OprD, OprG, and OprQ are involved in the uptake of small peptides, amino acids, or carboxylic acids (Hancock and Brinkman, 2002; Kucharska et al., 2015). OprE and OprC are induced in response to anaerobiosis and copper, respectively (Yoneyama and Nakae, 1996; Jaouen et al., 2006), and OprM is the OM component of the general efflux pump MexAB/OprM that is involved in general cell detoxification (Hancock and Brinkman, 2002). It should be noted that the abundance of these porins was found to be lower in bacteria that adhered to SS comparatively to PS and G, suggesting a reduction in OM permeability. A similar reduction in porin content has already been described in the OM of *P. aeruginosa* in response to a copper-induced stress (Teitzel et al., 2006), potentially suggesting a specific response to the metals composing the SS (iron, chromium, molybdenum, and nickel).

In addition to porins, other proteins associated with cell wall homeostasis were identified being produced at lower levels on SS than on the other two materials. This was the case for OprL, LptD, and PagL. OprL is homologous to the *Escherichia coli* Pal major OM lipoprotein that is involved in anchoring the peptidoglycan to the OM (Lloubès et al., 2001; Lim et al., 2012). LptD is an OM protein that is involved in the assembly of LPS in the OM outer leaflet (Srinivas et al., 2010). Lipid A 3O-deacylase PagL removes the 3-OH C10 or 3-OH C14 β-hydroxy fatty acid from lipid A, thus modifying the lipopolysaccharide structure (Ernst et al., 2006). Because lipid A is the biologically active component of LPS, its remodeling is known to alter the integrity of the bacterium's OM (King et al., 2009; Shah et al., 2013).

We therefore found on the one hand that numerous OM proteins were found at a lower abundance after adhesion to SS than to the other two surfaces. On the other hand, we were able to show that *P. aeruginosa* was able to adhere about 3- or 10-fold more to SS than to G or PS, respectively. This was quite surprising since it has been shown that some porins are involved in adhesion. Indeed, because they are exposed to the cell surface, some porins have been found to be involved in adhesion to biotic and/or abiotic surfaces. For example, this is the case for OprF (Azghani et al., 2002; Rebiere-Huet et al., 2002; Bodilis et al., 2004; Hemery et al., 2007; Fito-Boncompte et al., 2011), OprQ (Arhin and Boucher, 2010), and OprD (Paulsson et al., 2015). Taken together, these data enabled to highlight the importance of physicochemical properties to adhesion to solid surfaces. In *E. coli*, the low abundance of OM proteins including porins and the major lipoprotein Lpp, is correlated to a complex pathway called the general envelop stress response (ESR), which is under control of the master sigma alternative factor RpoE (Ades, 2004; Guo et al., 2014). RpoE maintains OM homeostasis by inducing synthesis of proteins involved in membrane repair and small regulatory RNAs (sRNAs) that down-regulate synthesis of abundant membrane porins and Lpp (Guo et al., 2014). Noticeably, it has been recently suggested that stress pathways, among which ESR, can play a role in surface sensing, leading to adhesion and biofilm formation (O'Toole and Wong, 2016). The periplasmic stress pathways Rcs (Regulator of capsule synthesis) and Cpx, which are members of the global ESR in *E. coli*, may indeed act as surface sensors with perturbation of the membrane(s), cell wall, and/or periplasmic space probably triggering these canonical stress systems, thereby perhaps alerting the microbe to cell-to-substratum contact (Otto and Silhavy, 2002; Morgenstein and Rather, 2012). In Enterobacteriaceae, specific Cpx pathway may prevent damages to the cell envelope caused by interactions of the cell with a surface (Otto and Silhavy, 2002). In *P. aeruginosa*, ESR is far less studied than in *E. coli*, and to date, only the two ECF sigma factors AlgU and SigX have been shown to be involved in maintaining the cell wall integrity (Wood and Ohman, 2009; Duchesne et al., 2013). Remarkably, the OM proteome of a SigX deficient *P. aeruginosa* strain was shown to present a much lower abundance in OM porins (Duchesne et al., 2013). Moreover, the conditions leading to cell wall stress among which membrane perturbations, were shown to increase SigX activity resulting in enhancing adhesion and biofilm formation (Bouffartigues et al., 2014, 2015). It may be conceivable that perception of an SS surface may occur *via* membrane or cell wall perturbations, leading to activation of the cell wall stress response of *P. aeruginosa*.

## Conclusion

This study showed that under our experimental conditions, the abiotic surface impacted the protein production of adherent cells of *P. aeruginosa* PAO1. About 7% of the proteome thus detected underwent quantitative changes that involved all cell compartments and particularly outer membrane proteins. To our knowledge, no such global proteomic study has previously been performed at such an early stage in the surface biocontamination process. This study showed that biological and physiological functions can be modified by the surface involved, suggesting that *P. aeruginosa* was able to sense the material to which it was adhering. However, the molecular mechanisms that lead to this surface sensing are probably numerous and complex, as highlighted by the various physiological responses of *P. aeruginosa* to adhesion on SS, G, or PS surfaces, and confirming the hypothesis that *P. aeruginosa* is capable of sensing and responding to a surface *via* specific programmes. These different proteomes found in *P. aeruginosa* adhering to surfaces with various physicochemical properties may also result in part from the different adhesion forces that exist between the surfaces of bacteria and materials. This physiological reactivity of bacteria to materials needs to be taken into account in sensitive environments where pathogenic strains may present a health threat, such as in hospitals and in the food industry. Further studies now need to investigate whether contact with the material can induce unwanted phenotypes for example cross-resistance to antibacterial products.

## Author contributions

Performed adhesion assays and protein extraction: MG, JB, and EB. Performed physicochemical characterizations: J-MH. Performed tandem mass spectrometry: AG. Performed protein quantification, heatmap representation, and statistical analyses: AG, AA and VM. Wrote the manuscript: all authors. Coordinated the study: M-NB-F.

### Conflict of interest statement

The authors declare that the research was conducted in the absence of any commercial or financial relationships that could be construed as a potential conflict of interest.

## Abbreviations

SS: stainless steel
G: glass
PS: polystyrene
SS/G: SS compared to G
SS/PS: SS compared to PS
PS/G: PS compared to G
RT: room temperature
R_a_: arithmetic average roughness
R_max_: maximum peak to valley height
SDS: sodium dodecyl sulfate
PseudoCAP: *Pseudomonas* Community Annotation Project
OM: outer membrane
OMP: outer membrane proteins
PagL: lipid A deacylase
QS: quorum sensing
SadC: surface attachment defective
Wsp: wrinkly spreader phenotype
LPS: lipopolysaccharide.
